# Salivary MRP-8/14 and the presence of periodontitis-associated bacteria in children with bonded maxillary expansion treatment

**DOI:** 10.1007/s00784-020-03706-6

**Published:** 2020-12-03

**Authors:** Michael Nemec, Nina Mittinger, Michael Bertl, Emanuela Liu, Erwin Jonke, Oleh Andrukhov, Xiaohui Rausch-Fan

**Affiliations:** 1 Division of Orthodontics, University Clinic of Dentistry, Medical University of Vienna, Vienna, Austria; 2 Division of Conservative Dentistry and Periodontology, University Clinic of Dentistry, Medical University of Vienna, Sensengasse 2A, 1090 Vienna, Austria

**Keywords:** Saliva, Orthodontic appliances, Oral health, MRP-8/14

## Abstract

**Objectives:**

The aim of this study was to investigate changes in saliva concentration of the inflammatory marker MRP-8/14 and the presence of some periodontitis-associated bacteria in patients with mixed dentition treated with a rigid acrylic, bonded maxillary expander.

**Methods:**

Fifteen patients in mixed dentition treated with a bonded palatal expander were enrolled in this longitudinal study. Saliva samples were taken before the therapy, as well as in 2 weeks and 3, 6, 9, and 12 months after the beginning of the therapy. In each sample, the levels of MRP-8/14 were determined by ELISA and the presence of 11 bacteria was detected by PCR followed by DNA-DNA hybridization.

**Results:**

Salivary concentration of MRP-8/14 and the amount of *Tannerella forsythia*, *Treponema denticola*, and *Eikenella corrodens* were significantly increased during treatment with bonded maxillary expander. These changes were transient and the maximal levels of MRP-8/14 and periodontitis-associated pathogens were observed 6–9 months after the beginning of the therapy.

**Conclusion:**

Therapy with bonded maxillary results in higher MRP-8/14 levels and increased prevalence of some periodontitis-associated bacteria, namely *T. forsythia*, *T. denticola*, and *E. corrodens*. The results suggest the detection of salivary MRP-8/14 levels may be a potential tool to reflect the oral health status in children with fixed orthodontic treatment.

**Clinical relevance:**

Our data suggest that the treatment with bonded maxillary expander might influence the oral health status and should be accompanied by the careful control of the oral health during the therapy.

## Introduction

The maintenance of oral health is mainly based on the host-microbial homeostasis in the oral cavity. Oral health might be disturbed during the application of different dental devices, particularly fixed orthodontic appliances, which are commonly used to correct malocclusions of various origins. Orthodontic treatment leads to modification and reorientation of periodontal tissues on the one hand, and the parts of orthodontic appliances may represent a reservoir for microorganisms and can affect oral microbiota and inflammation levels on the other hand [1, 2]. Additionally, orthodontic treatment with fixed appliances can also hinder oral hygiene due to restricted access to the tooth surface and subgingival area leading to a higher bacterial accumulation. The increased levels of pro-inflammatory cytokines may be due to the increased dental plaque accumulation or be induced by the increased mechanical loading [3, 4]. Clinically, gingivitis often occurs during orthodontic treatment due to impaired oral hygiene [5]. According to a meta-analysis, the level of subgingival periodontitis-associated bacteria increased temporarily after orthodontic appliance placement and returned to pretreatment levels several months later. This implies that fixed orthodontic treatment might not be causal for the periodontal disease [1]. Nevertheless, it remains still controversial whether orthodontic treatment can be considered as a risk factor in the pathogenesis of periodontal diseases.

Studies of the last decades focused on the salivary diagnostic of oral health status. The advantages of the saliva as the diagnostic fluid are none-invasiveness and the possibility to obtain the results quickly. Saliva has raised scientific and clinical importance in the detection of periodontal alterations [6]. Our previous study describes that the salivary content of periodontitis-associated bacteria appropriately reflects their presence in the subgingival plaques [7]. Salivary biomarkers can also be used to diagnose periodontitis and evaluate its severity [8]. Several proteins are currently considered as the potential biomarker of the inflammation and the early onset of periodontitis [9]. Myeloid-related protein-8/14 [10], also known as S100A8/9 or Calprotectin, is released under inflammatory conditions by polymorphonuclear leukocytes (PMNs), endothelial cells, monocytes, and macrophages [11, 12]. This calcium-binding protein is a sensitive inflammatory marker, and its levels in the gingival crevicular fluid are increased already in the early phases of experimental gingivitis [13]. Some studies show an increased level of MRP-8/14 in the gingival crevicular fluid and the saliva of the patients with periodontitis [14–16]. Furthermore, salivary MRP-8/14 levels correlate significantly with the clinical parameters of periodontitis and the presence of *Treponema denticola* [16].

Posterior crossbite sometimes occurs in the mixed dentition of growing Caucasians and is currently treated by maxillary expansion[17]. There are two types of fixed maxillary expansion appliances used for interceptive orthodontic treatment: bonded and banded [18, 19]. Rigid acrylic, bonded rapid maxillary expanders are commonly used to correct a maxillary transversal deficiency in mixed dentitions [20–22] and to open the maxillary sutures before treating maxillary skeletal deficiencies in growing children [23, 24]. Skeletal and dental effects and facial soft tissue changes after maxillary expansion are extensively reported in the literature [25–27]. However, the adverse impact of these appliances was also reported: a recent study shows that patients wearing full-coverage bonded rapid maxillary expanders developed after two months more white spot lesions than the control group [28]. However, there still is a lack of evidence concerning inflammatory processes and bacterial load in patients with long-term wear of these appliances covering major parts of the teeth surfaces.

Our prospective clinical trial aimed to investigate the changes in salivary levels of MRP-8/14 and bacteria associated with periodontal disease in children with mixed dentition treated with a rigid acrylic, bonded rapid maxillary expander up to 12 months.

## Material and methods

### Patients’ selection

The present prospective study included fifteen children from 8 to 10 years old (six boys and nine girls, mean age: 9.0 ± 0.9 years) with posterior crossbite treated with bonded acrylic maxillary expanders. All study participants were patients of the Division of Orthodontics at the University Clinic of Dentistry, Medical University of Vienna, from 09/2013 to 02/2016. Clinical examination was performed, and treatment planning was conducted by the staff of the Division of Orthodontics.

At the beginning of orthodontic treatment, oral hygiene of all participants was controlled routinely, and lack of sign of gingival and periodontal inflammation was recorded intraorally and on panoramic radiographs. Exclusion criteria were defined as follows: systemic disease, impaired nose breathing, allergic reaction to tartrazine or benzoic acid, trauma or bleeding in the oral cavity, intake of antibiotics, or use of antiseptic mouth rinse three months before enrollment to the study, apparent gingival or periodontal disease.

The study was approved by the ethical committee of the Medical University of Vienna, Austria (1678/2013). All participants and their parents were thoroughly informed about the aims and methods and gave their written consent. The study was conducted in accordance with the Helsinki Declaration as revised in 2013.

### Bonded maxillary expander placement

**Fig. 1:**
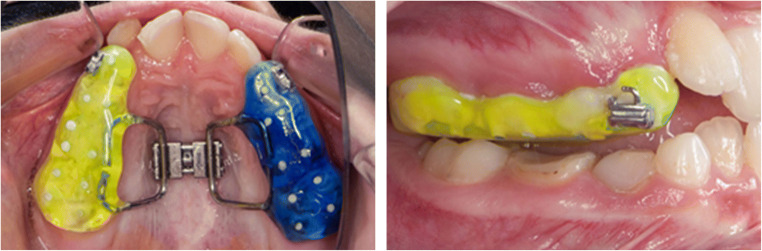
Bonded maxillary expander of hyrax type. Acrylic plates covering the teeth surfaces of the deciduous teeth 3, 4, and 5 and the first molar in the upper arch. The appliance was bonded with glass ionomer cement

A conventional bonded maxillary expander was used in the present study. This orthodontic appliance consists of two indirectly manufactured acrylic plates covering the teeth surfaces of the deciduous teeth 3, 4, and 5 and the first molar in the upper arch (Fig. 1). Both acrylic plates were connected by a palatal screw of the hyrax type. After controlling the fit of the appliance, the appliance was bonded with glass ionomer cement to the teeth. Ketac™ Cem Glass ionomer cement (3 M, Neuss, Germany) excess was removed, and the expander remained cemented for 12 months [20].

### Oral hygiene protocol

Patients and parents received detailed oral hygiene instructions at the beginning of treatment. Patients were trained in home oral hygiene with manual tooth brush, to prevent leaching out of cement. Especially in the region of the flanking lateral upper incisor, patients were instructed in the use of floss. Oral hygiene was checked on every appointment and further oral hygiene instructions were given, if needed. Due to the material thickness and properties of the acrylic plates and their close location to the gingiva, no periodontal probing or plaque monitoring was feasible.

### Saliva collection and MRP-8/14 analysis

The collection of stimulated whole saliva was performed directly before the bonding of the appliance and after 2 weeks and 3, 6, 9, and 12 months after bonding, all measurements took place in situ with the bonded appliance. Participants were asked to refrain from eating 2 h before the bonding of the appliance and sampling. Saliva was collected using a saliva collection system (Greiner Bio-One, Kremsmuenster, Austria), as described in our previous studies [16, 29]. First, participants had to rinse their mouth for 1 min with water. After that, patients kept in their mouth 4 ml of a saliva extraction solution containing food coloring E102 (Tartrazine) for 2 min. This solution stimulated the salivary flow for stimulated whole saliva collection. The collected mixture of saliva and extraction solution was immediately transferred to tubes containing sodium azide against microbial growth and centrifuged (3220 rpm, 4 °C). Approximately 2 ml of this mixture was left in the transfer tube and frozen at − 40 °C until microbial analysis. Collected samples were centrifuged immediately for 10 min at 3220 rpm at 20 °C. The supernatants were transferred into transfer tubes containing 4 mg of the preservative benzoic acid, aliquoted, and frozen at − 80 °C until analysis.

The remaining pellet in the transfer tube was stored at − 20 °C and used for the microbiology analysis.

Quantitative detection of MRP-8/14 in the samples was performed by commercially available ELISA (Bühlmann Laboratories AG, Schoenbuch, Switzerland) according to the manufacturer’s protocol as previously described [16]. Since the collected samples included stimulated saliva and extraction solution, the saliva percentage was measured photometrically using a saliva quantification kit (Greiner Bio One, Kremsmuenster, Austria). The salivary levels of MRP-8/14 were calculated by multiplying its content in the samples with the relative content of saliva in the samples.

### Detection of bacteria associated with periodontal disease

The pellet obtained after centrifugation of stimulated saliva was used for the microbiological analysis. Eleven different bacterial species were identified by commercially available kit (micro-IDent® plus 11-test, Hain Lifescience, Nehren, Germany). This kit is considered as a reliable tool for the microbiological diagnostic of periodontal bacteria [30, 31]. The detection procedure included DNA isolation, amplification of specific DNA by PCR, and detection with specific DNA-probe by DNA-DNA hybridization. The following bacteria were detected: *Aggregatibacter actinomycetemcomitans*, *Porphyromonas gingivalis*, *Prevotella intermedia*, *Tannerella forsythia*, *Treponema denticola*, *Parvimonas micra*, *Fusobacterium nucleatum*, *Campylobacter rectus*, *Eubacterium nodatum*, *Eikenella corrodens*, and *Capnocytophaga spp.* (*C. gingivalis*, *C. ochracea*, and *C. sputigena*). The microbiologic results were semi-quantitative and scored as −, (+), +, ++, and +++ according to a colorimetric index. For all species excepting *A. actinomycetemcomitans*, bacterial load was categorized to the following logarithmic steps: < 10^4^, 10^4^, < 10^5^, < 10^6^, > 10^7^. The data for *A. actinomycetemcomitans* were categorized as follows: < 10^3^, 10^3^, < 10^4^, < 10^5^, > 10^6^.

### Statistical analysis

For descriptive analysis, median and quartiles were calculated. Differences in the salivary levels of MRP-8/14 and bacterial load at different times after the treatment compared to the initial time point were calculated using a non-parametrical Wilcoxon test. Statistical analysis was performed using a statistical program (SPSS 23.0; IBM, Armonk, NY). Power analysis was done using the online tool (HyLown Consulting LLC, Atlanta, GA). To calculate statistical power for comparing salivary MRP levels between different time points, the differences of the corresponding values were calculated. The mean values and standard deviations were then calculated for these differences and were further used to calculate statistical power using the procedure “1 mean, 1-sided.” The level of statistical significance was set at *p* < 0.05 and statistical power of 80%. The level of statistical significance was set at *p* < 0.05 and a statistical power of 80%.

## Results

### Drop-out quotes

All parameters were recorded before the therapy in 15 study participants, in 2 weeks after the beginning in 13 participants, in 3 months in 12 participants, in 6 months in 14 participants, and in 9 and 12 months in 12 participants. This dropout was caused by the relocation of the patient’s family in one case and missed appointments by the patients in other cases.

### Salivary levels of MRP-8/14 in children during bonded maxillary expansion treatment

**Fig. 2:**
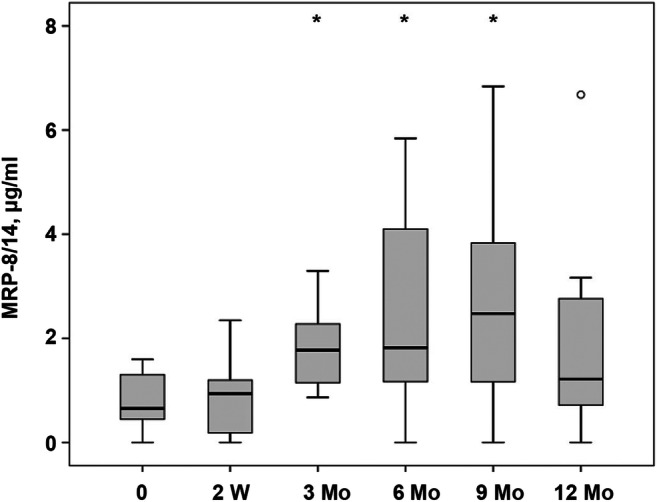
Salivary MRP-8/14 levels in children in the course of bonded maxillary expansion treatment. The levels of MRP were measured in stimulated whole saliva before the therapy (“0”) and at the different time points after the beginning of therapy. Data are presented as box (25–75th percentiles) and whisker (10–90 percentiles) plots. The horizontal line inside the box indicates the median (the 50th percentile). An asterisk indicates significantly different compared to the values before the therapy (Wilcoxon test)

Figure 2 shows the salivary levels of MRP-8/14 in children with bonded maxillary expansion before the therapy, and at different time points after the beginning of the treatment. The salivary levels of MRP-8/14 have gradually increased during the treatment for up to 9 months after the start of therapy. The levels of MRP-8/14 after 3, 6, and 9 months after therapy start were significantly higher compared to the initial MRP-8/14 levels. The statistical power was calculated to be 97%, 97%, and 98% for 3, 6, and 9 months, respectively. After 12 months of therapy, some recovery of salivary MRP-8/14 was observed, and no statistical difference compared to the initial point was detected.

### Presence of periodontitis-associated bacteria in children saliva in the course bonded maxillary expansion treatment

**Fig. 3:**
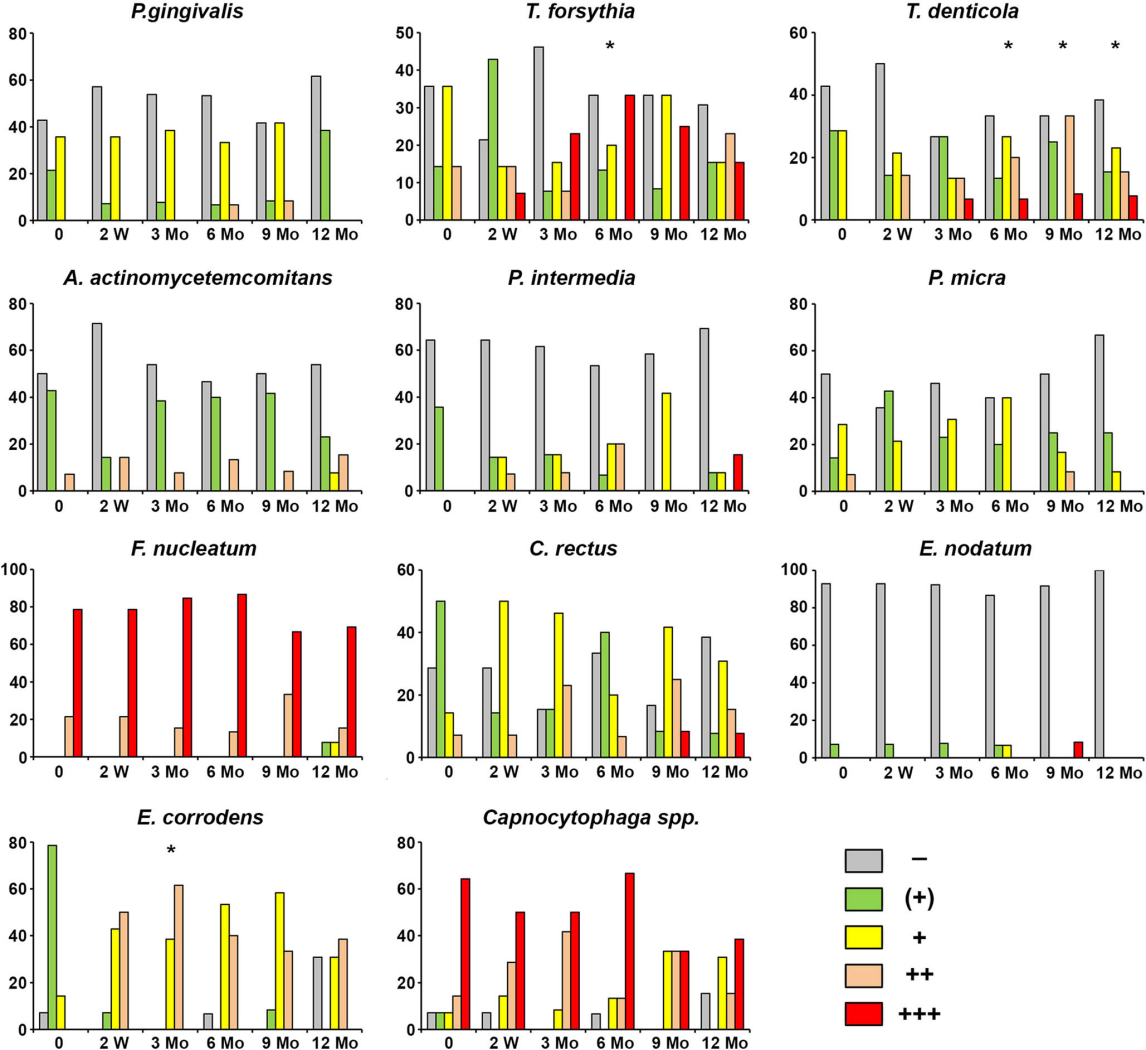
The presence of periodontitis-associated bacteria at six different time points. The level of different bacteria measured in the stimulated saliva using DNA hybridization technology. *Y*-axis shows the percentage of individuals with the given detection levels of certain periodontitis-associated bacteria at the different time points. An asterisk indicates statistically higher presence compared to the beginning of the therapy

The detection frequency and the quantities of various bacteria in the course of bonded maxillary expansion treatment are summarized in Fig. 3. The occurrence rate and amount of some periodontitis-associated bacteria tended to increase in the course of therapy. Compared to the initial time point, a significantly higher amount was observed for *T. forsythia* in 6 months after therapy beginning, for *T. denticola* in 6, 9, and 12 months after therapy beginning, and for *E. corrodens* in 3 months after therapy beginning. No significant differences for other bacteria and time points were observed.

## Discussion

The present study investigated the changes in the salivary concentration of the inflammatory marker MRP-8/14 and periodontitis-associated bacteria in patients with mixed dentition treated with a rigid acrylic, bonded maxillary expander. Saliva collection represents an uncomplicated and straightforward method [32], which can be easily used as a diagnostic tool for children. MRP-8/14 has been suggested as a potential indicator of disease activity in periodontitis patients [13, 33] and a diagnostic marker for monitoring periodontal treatment [34]. The present study at first time demonstrates the levels of MRP-8/14 in the saliva of children with bonded maxillary expanders and assesses its relationship with a local bacterial load.

We found that the salivary MRP-8/14 levels in children were increased up to two times in the course of therapy. MRP-8/14 is a very sensitive inflammatory marker because its local level is significantly increased immediately after the cessation of oral hygiene in the experimental gingivitis model [13]. Our previous studies show that salivary periodontitis patients exhibit two to three times higher salivary MRP-8/14 levels than healthy controls [16, 35]. Thus, quantitatively similar changes in the salivary MRP-8/14 levels are observed in periodontally healthy children with maxillary expanders and the patients with advanced periodontitis. A recent report shows that salivary MRP-8/14 levels are increased by about two times in gingivitis and periodontitis patients compared to the healthy controls [36]. Thus, MRP-8/14 is increased at the early inflammation stages and could be considered as an important marker for oral health status.

Microbial detection of bacteria associated with periodontal disease in the saliva of young patients has been shown in previous studies [37–41]. Sakai et al. detected putative periodontitis-associated bacteria in the saliva of children with mixed dentition and healthy periodontal conditions. They reported a prevalence of *T. denticola* between 50 and 71.9% [38]. Eick et al. show that despite periodontitis-associated bacteria were detectable in Swiss adolescents between 15 and 18 years, there were no clinical signs of periodontitis, but only those of gingivitis [41]. There is no hard proof that colonization with periodontal bacteria in children will lead to periodontal disease similar to that in adults; however, the earlier onset of the infection might enhance the probability of permanent colonization [39] of the oral cavity. Our study demonstrated that treatment with a bonded maxillary expander in patients without clinical signs of periodontal inflammation before treatment affected the presence of some periodontitis-associated bacteria in the saliva of children with mixed dentition. Particularly, a significant increase in the presence of *T. denticola* and *T. forsythia*, two out of three members of red-complex bacteria, which are strongly associated with periodontitis, was detected. Besides, an increased presence of *E. corrodens* in the course of the therapy was observed. Periodontitis-associated bacteria, and particularly red-complex bacteria, do not belong to the healthy oral microbiome. These strictly anaerobic species usually appear only at the later stages of biofilm formation. Their appearance can be explained by the impaired access of some places in the oral cavity during tooth brushing and, consequently, disruption of oral hygiene during the treatment. Treatment with bonded maxillary expanders might also lead to an increase in caries-associated species. Particularly, a previous study reports significantly higher levels of *Streptococcus mutans* and *Lactobacillus spp.* in patients treated with bonded maxillary expanders after six months of treatment [42].

Alteration of the mouth colonization by oral bacteria is expected during orthodontic treatment. Various studies have been performed on the effect of fixed orthodontic appliances on microbiological and clinical parameters [43, 44]. It is known that orthodontic appliances influence the accumulation of plaque and the colonization with periodontitis-associated bacteria leading to inflammatory conditions [45]. Gujar et al. could show by checkerboard DNA-DNA hybridization that most bacterial species on orthodontic appliances showed moderate counts during treatment of a relatively young patient collective (ranging 11 to 29 years) with fixed labial, fixed lingual, and aligner appliances. *T. forsythia* counts were moderate in all three appliances, whereas *T. denticola* showed higher bacterial counts in all three different orthodontic appliances than other bacterial species [46]. Our findings support the above reports. Still, the objects we observe are children 8–10 years old under treatment with bonded maxillary expander.

The changes in oral health in patients with maxillary expanders have a somewhat transient character. We have observed a tendency that MRP-8/14 values were higher during maxillary expansion treatment in comparison to the beginning and end of treatment. The maximum values of MRP-8/14 could be detected at 6–9 months after bonding, and these values were higher than those at 12 months after bonding. It seems that the treatment leads to the transient worsening of oral health. Similar transient changes in the oral microbiota and the level of inflammatory cytokines are reported for the other orthodontic treatments [47]. The initial worsening in oral health after the beginning of the therapy could be due to impaired oral hygiene, leading to an alteration in the bacterial colonization and consequently increased host response. However, after a certain time, the immune system may adapt to the changed bacterial load, resulting in the diminished host response and the recovery of salivary MRP-8/14 levels to those similar at the beginning of the therapy. Improved oral hygiene procedures, especially immediately after the starting the orthodontic treatment, might be important to maintain the oral health in these patients.

A potential limitation of our study can be a rather low number of enrolled patients. This could be explained by the rather strict inclusion criteria and generally low number of patients treated with this therapeutic modality. Similarly to our study, a previous report on a similar patient group included only 16 patients [28]. Another study of surgically assisted rapid palatal expansion in adult patients on periodontal effects included 14 patients (9 females, 5 males) treated by a bonded Hyrax-type expander [48]. Despite the rather low participants’ number, the differences detected in our study reached the statistical power of 80%. Therefore, the results of our pilot study might be clinically relevant and should be confirmed in further studies with higher numbers of participants and other inflammatory markers.

Another limitation is a relatively low number of detected bacteria. The oral cavity has a very complex microbiota consisting of up to 700 bacterial species [49, 50]. Therefore, the determination of the only 11 species does reflect the changes in the oral microbiota upon the orthodontic treatment. Moreover, we have measured only bacterial DNA, which does not give information about bacteria’s viability and function. Oral health is largely based on the homeostasis between oral microbiota and the host immune system, which depends on numerous host-derived and ecological factors [51]. The introduction of a new device in the oral cavity and its cementation provide new surfaces for bacterial attachment and limit oral hygiene. Thus, it is an essential ecological factor influencing host-microbial homeostasis and cause dysbiosis. Most of the investigated bacteria are associated with periodontal disease, and their appearance or increased presence could suggest by the shift of the oral ecosystem to a dysbiotic state [52]. Therefore, in future studies, the alteration of the oral microbiome during orthodontic therapy should be investigated by omics technologies [53]. It would be necessary to study not only the composition of the microbial community using 16S rRNA but also the alteration of the functional activity by metatranscriptome analysis [54].

The last possible point of criticism could be that we measured only the absolute concentration of MRP-8/14. The concentration of various salivary components depends on the salivary flow, which exhibits a large inter-individual variability [55]. Such factors as using the stimulated saliva and the longitudinal study design might partially eradicate the inter-individual differences in the salivary flow.

## Conclusion

According to the existing literature, this is the first study indicating that treatment with bonded maxillary expander results in the increased salivary levels of the salivary marker of periodontitis MRP-8/14. Moreover, this therapeutic modality resulted in the increased presence of some periodontitis-associated bacteria like *T. forsythia*, *T. denticola*, and *E. corrodens*. Our data suggest that the treatment with bonded maxillary expander might influence oral health status and should be accompanied by the careful control of the oral health during the therapy.
